# Crucial Roles of microRNA-Mediated Autophagy in Urologic Malignancies

**DOI:** 10.7150/ijbs.61175

**Published:** 2021-07-31

**Authors:** Maolei Shen, Xin Li, Biao Qian, Qiang Wang, Shanan Lin, Wenhao Wu, Shuai Zhu, Rui Zhu, Shankun Zhao

**Affiliations:** 1 Department of Urology, Taizhou Central Hospital (Taizhou University Hospital), Taizhou, 318000, Zhejiang, China.; 2 Department of Urology, the First Affiliated Hospital of Gannan Medical University, Ganzhou, Jiangxi, China.; 3 Department of Thoracic Surgery, Taizhou Central Hospital (Taizhou University Hospital), Taizhou, 318000, Zhejiang, China.; 4 School of Medicine, Taizhou University, Taizhou, 318000, Zhejiang, China.; 5 Department of Cardiovascular Surgery, The Second Affiliated Hospital of Zhengzhou University, Zhengzhou, 450014, Henan, China.

**Keywords:** MicroRNA, Autophagy, Molecular mechanisms, Urologic oncologies

## Abstract

Urologic oncologies are major public health problems worldwide. Both microRNA and autophagy, separately or concurrently, are involved in a variety of the cellular and molecular processes of multiple cancers, including urologic malignancies. In this review, we have summarized the related studies and found that microRNA-mediated autophagy acted as carcinogenic factors or suppressors in prostate cancer, kidney cancer, and bladder cancer. MiRNAs, targeted genes, and the different signaling pathways constitute a complex network that orchestrates autophagy regulation, militating the oncogenic and tumor-suppressive effects in urologic malignancies. Aberrant expression of miRNAs may induce the dysregulation of the autophagy process, resulting in tumorigenesis, progression, and resistance to anticancer therapies. Targeting specific miRNAs for autophagy modulation may present as reliable diagnostic and prognostic biomarkers or promising therapeutic strategies for urologic oncologies.

## Introduction

Urologic oncology mainly includes prostate cancers, bladder cancers, kidney cancers, adrenal tumors, penile neoplasms, testicular neoplasms, and ureteral neoplasms. According to the GLOBOCAN 2018, the incidence and mortality of urologic oncologies are reported at 12.3% and 7.7%, respectively 1. Prostate cancer (PCa) is the most common malignancy and the second leading cause of cancer-related death among male subjects in Western countries. As reported, bladder cancer is the 10^th^ most common cancer worldwide in 2018 1. Renal cell carcinoma is one of the most frequently diagnosed cancers, representing 2-3% of all malignant tumors in adults. As predicted, the incidence of urological cancer may elevate substantially amid a growing, aging population. As a result, examinations and interventions of urologic cancers may continuously bring a large financial burden worldwide. Statistics showed that in 2020, the annual costs for treating and curing prostate, bladder, and kidney cancers in the United States were projected to reach $31.47 billion 2.

Despite recent advances in diagnosis and therapy for urologic oncologies, these cancers, especially in patients with advanced and metastatic conditions, are still the leading causes of death as compared to other urologic diseases. Radical surgery is still the first therapeutic option for the early stage of urologic cancers. However, unresectable patients with advanced-stage of tumors usually have a poor prognosis due to their highly resistance to chemotherapy or radiotherapy.

Exploring the molecular mechanism of tumorigenesis in urologic oncologies would be a prerequisite for the diagnosis, therapeutic management, and prognosis of these diseases. Finding a way to increase the sensitivity of radiotherapy and chemotherapy for urologic cancers is urgent. It is well known that cell apoptotic metabolic disorders play a key role in multiple cancers, including urologic cancers 3-5. A growing number of studies demonstrated that microRNAs (miRNAs) could promote apoptosis of cells by regulating autophagy 6-8. In this review, we aim to systematically review the relevant literatures to characterize the effect of the miRNAs-autophagy axis in the progression and prognosis of human urologic oncologies.

## Overview of Autophagy

Autophagy is characterized by “self-digestion”, which is an effective cellular process toward maintaining cellular biosynthesis and energy requirement for the eukaryotic cells. So far, there are three main subsets of autophagy, including macroautophagy, microautophagy, and chaperone-mediated autophagy (CMA) 9. The most commonly investigated autophagy usually indicates macroautophagy. It begins with the formation of the endoplasmic reticulum and gradually expands into the precursor of the autophagosome, i.e., isolation membrane or cup-shaped phagophore 10. The autophagy microtubule-associated protein light chain 3 (LC3) forms LC3-Ⅰ and LC3-Ⅱ through a series of chemical reactions, which ensure elongation and expansion of autophagy 11. Next, cytoplasmic components are engulfed with phagophore and sealed into a double-membrane vesicle, termed the autophagosome 12. Autophagosome fuses with the acidic lysosomal membrane, forming autolysosomes, where autophagic body together with its cargo are degraded 12. Autophagy is a highly conserved catabolic process in which cellular unnecessary or dysfunctional materials (such as mitochondria and proteins) are transported to the lysosome for degradation 13. Autophagy-related genes (ATGs), first been identified in yeast and later found in mammals, were subsequently proved to being mediated by the autophagosome formation 14. Under stress, autophagy exerts a cytoprotective effect by eliminating damaged organelles and proteins. Conversely, hyperactivation of autophagy was shown to induce autophagic cell death. Autophagy has dual roles in both oncogenicity and tumor suppressor according to different molecular mechanisms.

## The role of autophagy in urological cancers

Mounting evidence demonstrates that dysregulation of autophagy may correlate with numerous human diseases. In 1999, Levine* et al.* 15 first reported the association between autophagy and tumor. However, the exact role of autophagy on multiple cancers remains a debate. Many investigators suggest that autophagy may have an antitumorigenic effect but quite a few researchers believe autophagy can promote tumorigenesis and the progression of cancers. For urological tumors, it was reported that autophagy served as a tumor suppressor by maintaining genomic integrity. Once a tumor has been established, autophagy can be utilized by cancer cells to survive cellular stresses in the unfavorable microenvironment. Poillet* et al.* 16 demonstrated that autophagy might induce epithelial-mesenchymal transition (EMT) in bladder cancer via the TGF-β1/Smad3 signaling pathway, which significantly promoted the invasion of the cancer cells. As reported, the level of autophagy is low in normal cells but increase in numerous cancer cells due to the elevated metabolic demand of cancer cells 17, 18. Autophagy provides bioenergetic and biosynthetic substrates to the TCA cycle by recycling macromolecules, which can maintain energy homeostasis 19, 20. Therefore, autophagy may promote tumor growth and survival through its capability to sustain metabolic functions of tumor cells.

Autophagy defects have been identified in prostate cancer, bladder cancer, and kidney cancer, which indicate that it is also a tumor suppressor in these tumors 21-23. Paradoxically, it was also reported that elevated autophagy could promote the progression of urinary tumors. Lu* et al.* 24 suggested that increased expression of autophagy induced by dCTP pyrophosphatase 1 was related to unfavorable outcomes of prostate cancer. Tong *et al.* 25 demonstrated that a high level of autophagy induces EMT via the TGF-β1/Smad3 signaling pathway, which significantly promotes the invasion of bladder cancer cells. Inversely, chloroquine (CQ) or 3-methyladenine (3MA) remarkably decreased EMT-mediated invasion by inhibiting autophagy 25. A more recent study developed by Patergnani *et al.* 26 revealed that autophagy increased both cell proliferation and migration of kidney cancer cells by degrading p53. Moreover, they further found that the capability of both proliferation and migration was significantly inhibited by suppressing the expression of the autophagy, while p53 degradation was reduced 26. Based on the above evidence, autophagy is closely related to the progression of urinary tumors. Therefore, it is very important for the treatment of urinary tumors to control their expression.

Autophagy can be regulated by many proteins. The mammalian target of rapamycin (mTOR) and the AMP-responsive protein kinase (AMPK) pathways are considered to be major pathways involved in autophagy 27. Under nutrient-rich conditions, mTOR is activated and inhibits autophagy by phosphorylating unc-51-like kinase 1(ULK1), a key protein for inducing autophagy. On the contrary, nutrient deprivation stimulates autophagy via stimulating AMPK to activate Tuberous Sclerosis Complex (TSC1/2), the suppressors of mTOR 28. On the other hand, several cancer-linked genes, (p53, p62, p21, STAT3, and BCL2) and tumor-associated stressful signals (MEK/ERK, IRE1/JNK, PERK/eukaryotic initiation factor 2α (eIF2α)/ ATF4 pathway) also stimulate or inhibit autophagy 29.

In addition to various autophagy-related proteins, autophagy also tightly interplays with microRNAs (miRNAs) and miRNAs triggered signaling. Extensive studies have revealed that mountains of microRNAs are involved in the regulation of autophagy.

## miRNAs

MiRNAs are a major class of conserved and single-stranded noncoding RNAs found in a wide range of animals, plants, and some viruses, which play essential roles in post-transcriptional gene silencing by promoting messenger RNA (mRNA) degradation or by inhibiting mRNA translation 30. Most miRNAs genes are transcribed as large primary miRNAs. They contain a few stem-loop structures which consists of approximately 70 nucleotides each 30. Mature miRNAs are guided to the 3 end of their target mRNA, and contributions to the mRNA translational repression. The first miRNA was found in Caenorhabditis elegans in 1993 and the first human miRNA in 2000 31. Currently, 2,675 human mature miRNAs have been identified in the miRBase miRNA database. However, the biology of miRNAs is very complex. Presently, many molecular mechanisms of miRNA activity have been uncovered, such as single nucleotide polymorphisms, asymmetric miRNA strand selection, histone or DNA methylation, and RNA editing 32. They tightly regulate the biological processes including differentiation and cell proliferation under physiological conditions or in diseases 32. Numerous physiological and pathological processes, including cancer, metabolic and cardiovascular diseases, relying highly on miRNAs.

The expression levels of miRNAs in human cancers are different from that in healthy cells. The relationship between miRNA expression and cancer development has been described for the first time in chronic lymphocytic leukemia 33. It was reported that miRNAs have a two-sided effect on cancer.

Cancer-associated miRNAs are correspondingly divided into oncogenic miRNAs (oncomiRs) and tumor-suppressive miRNAs (miRsupps). OncomiRs contribute to tumor progression and are frequently highly expressed in cancer cells 34. MiRsupps inhibit tumorigenesis by regulating cell growth, apoptosis, and other cancer-associated events, and these are usually down-regulated in various cancers 34. Studies also showed that single miRNAs can play different roles in different cancers. Yang *et al.* 35 discovered that the upregulated miR-17 significantly inhibits cell proliferation, migration, and invasion abilities in bladder cancer cells. Ting *et al.* 36 reported that b-cell lymphoma patients with increased miR-17 expression had a shorter progression-free survival, indicating it plays an oncogenic role.

Recently, more and more studies have shown that some regulators at induction, vesicle nucleation, vesicle elongation, and retrieval stages of autophagy can be regulated by miRNAs. Zhu *et al*. 37 first confirmed that miR-30a could markedly negatively regulate the autophagic activity. Soni *et al*. 38 demonstrated that in breast cancer cells, miR-489 could reduce tumor cell survival through inhibiting autophagy by targeting ULK1 and sensitizes tumor cells to doxorubicin via autophagy inhibition.

Zhang *et al*. 39 have proved that MicroRNA-133a-3p could block autophagy-mediated glutaminolysis by targeting ATG13, further inhibiting gastric cancer growth and metastasis. MiRNAs also emerged as key regulators for autophagy in urologic cancers. This led us to review related literature to identify the regulation of autophagy by miRNAs associated with urologic cancers, which may contribute to developing new therapeutic strategies to target urologic cancers effectively.

## Regulation of autophagy by miRNA in urologic cancers (Table 1)

### Interaction of miRNA and autophagy in PCa (Figure 1)

#### MiR-361-5p/Sp1/PKM2 signaling

In the autophagy and miRNA literature, the most widely studied urologic cancer is the PCa. MiR-361-5p acts as an anticancer role in PCa by regulating autophagy 40, 41. Ahmad *et al.* 42 reported that pyruvate kinase isoenzyme type M2 (PKM2) affected the autophagic process by upregulating LC3B or Beclin-1. Studies have shown that specificity protein 1 (Sp1) directly regulates PKM2, controlling the autophagic process 43. Sp1 plays a key role in PCa progression by regulating cell proliferation, angiogenesis, apoptosis, migration, and invasion 44. It is reported that Sp1 knockdown significantly decreased the expression levels of PKM2, and inhibited autophagy and cell growth in PCa 45. Recently, Ling *et al.* 45 reported that highly expressed miR-361-5p in PCa cell lines negatively regulates Sp1 and PKM2 by directly targeting the binding site in the 3' untranslated region (3'UTR), subsequently affecting the autophagic process. Meanwhile, it dramatically suppresses PCa cell growth and migration 45. Notably, miR-361-5p inhibits autophagy by suppressing the Sp1/PKM2 signaling, consequently affecting the proliferation and metabolism of PCa cells, which is a potential target in PCa therapy.

#### MiR-143/autophagy-related 2B (ATG2B) signaling

It was reported that curcumin (diferuloylmethane) had a promising anti-tumor effect in multiple cancers 46. Curcumin might suppress damage-induced autophagy in various cancer cells. Besides, curcumin is considered to be a radiosensitizer in PCa. miR-143 is well-identified being a tumor suppressor in some types of cancers, including PCa. Liu et al. demonstrated that miR-143 could inhibit autophagy in PC3 and DU145 cells via downregulating ATG2B expression, leading curcumin sensitizes PCa cells to radiation 47.

#### MiR-34a, miR-381, and miR-146b mediated mTOR signaling

miR-34a has been frequently identified as a tumor suppressor or oncogene and deregulated across different cancer types. As is known, mTOR is a serine/threonine kinase that regulates varieties of cellular processes which include autophagy 48. It is reported that mTOR inhibitors were used as immunosuppressors and be approved for the treatment of malignancies 48. miR-34a was downregulated in PCa and overexpression of it reduced proliferation and colony formation 49. Liao *et al*. 50 reported that miR-34a overexpression significantly downregulated p-AMPK and upregulated p-mTOR, which inhibited autophagy and enhanced chemosensitivity in PCa.

A more recent study 51 indicated that over-expression of miR-381 could inhibit the PI3K/AKT/mTOR signaling pathway by down-regulating reelin (RELN), resulting in promoting PCa cell apoptosis. The author further concluded that miR-381 might function as a tumor suppressor for PCa and speculated this biological effect of miR-381 was depended on the strengthening of autophagy.

On the contrary, a study developed by Gao et al. 52 demonstrated that upregulation of miR-146b promoted the proliferation of PCa cells by activating the AKT/mTOR signaling pathway. However, they observed that the autophagy process was enhanced after elevating the miR-146b expression, which was opposite with the functioning of miR-34a/mTOR.

Collectively, miR-34a, miR-381, and miR-146b could affect PCa progression by regulating mTOR-related autophagy but the exact mechanisms were different among studies.

#### MiR-301a/miR-301b/NDRG2 signaling

MiR-301a and miR-301b are two oncogenes involved in multiple cancers, including PCa. Previous studies indicated that the expression of miRNA-301a/b was evidently higher in PCa than in the normal prostate tissues 19, 53. Further study showed that miRNA-301a/b promoted cell proliferation and autophagy of PCa cells 19, 53. It was reported that n-myc downstream regulator gene 2 (NDRG2), a member of the alpha/beta hydrolase superfamily, might be involved in the regulation of autophagy and is also considered to be a tumor suppressor in PCa 54. A study from Wang *et al.* 55 showed that miRNA-301a/b can bind to 3'UTR of NDRG2 and significantly downregulate its expression, subsequently enhancing autophagy and radioresistance in PCa cells. Additionally, they also observed that autophagy and radioresistance of PCa cells were markedly enhanced after knockdowning NDRG2, while highly expressed NDRG2 can inhibit autophagy and promote radiosensitivity 55. Based on this evidence, miR-301a/b-NDRG2 axis might be a key signaling pathway regulating the radiosensitivity of PCa cells.

#### MiR-32/DAB2 interacting protein (DAB2IP)

MiR-32 functioned as an oncomiR in PCa, breast cancer, and colorectal carcinoma, which could modulate the tumor growth and metastasis. DAB2 interacting protein (DAB2IP) is also called aspartokinase (ASK1)-interacting protein-1 and is downregulated, with autophagy inhibitory and apoptosis enhancing, in PCa cells 56. Liao *et al*. 57 reported that miR-32 overexpression significantly inhibited DAB2IP expression via a directly binding site within the DAB2IP 3'UTR in PCa. In addition, both miR-32 mimics and DAB2IP-knockdown dramatically promote cell survival and decrease radiosensitivity in PCa cells 57. More significantly, autophagy was significantly enhanced by overexpression of miR-32 and knockdown of DAB2IP 57. On the basis of the above reports, miR-32 may have an important effect on the radioresistance by suppressing autophagy through targeting DAB2IP in PCa and may provide a therapeutic target for treating PCa.

#### MiR-124/miR-144/polymers of intrinsic microporosity-1 (PIM1) signaling

It was reported that miR-124 and miR-144 might serve as tumor suppressors. Up-regulation of miR-124 and miR-144 significantly could inhibit the proliferation, migration, and invasion of PCa cells 58, 59. The PIM kinases are the family of serine/threonine kinases and involved in regulating proliferation, apoptosis, metabolism, and autophagy of cancer cells 60. PIM1 is an important subtype of it. Eerola *et al.* 61 found that the expression of PIM1 is usually upregulated in PCa, which promoted the capability of PCa cells migration and invasion. Gu *et al.* 62 confirmed that miR-124 and miR-144 might simultaneously regulate PIM1 by binding to their 3'UTR. PIM1 overexpression significantly enhanced autophagy and reduced apoptosis after irradiation. Paradoxically, PIM1 knockdown reduced autophagy and enhance the sensitivity PCa cells to irradiation. On the basis of the above evidence, overexpression of miR-124 and miR-144 may inhibit autophagy and enhance radiosensitivity by downregulating PIM1 in PCa.

#### MiR-26b/unc-51 like kinase 2 (ULK2) axis

So far, mounting evidence showed that miR-26b played a key role in the development of multiple cancers, including PCa 63. Hodzic *et al.* 64 reported overexpression of miR-26b significantly augmented PCa cell death, which suggests its inhibitory effect on the tumor. Wang *et al*. 65 found that ULK2 can interact with Atg13 and FIP200 to stimulate autophagy, subsequently, modulating the proliferation and apoptosis of tumor cells. A further study from John *et al*. 66 found that miR-26b can inhibit autophagy by targeting ULK2 in PCa cells, promoting cell apoptosis. However, ULK2 overexpression dramatically rescued miR-26b mediated autophagy inhibition in PCa cells 66. In summary, miR-26b/ULK2 has a modulatory effect on the development of PCa and may be a novel therapeutic target for PCa.

#### MiR-212/Sirtuin 1 (SIRT1) signaling

MiR-212, a non-coding RNA located in chromosome 17p13.3, has both tumor-promoting or tumor-suppressor functions in multiple cancers 67. It has been reported to be derived from an intron of a non-protein-coding gene. A recent study has demonstrated miR-212 is significantly down-regulated in PCa as compared to the normal epithelium and/or stroma 68. Moreover, a study showed that miR-212 overexpression dramatically suppressed PCa cell proliferation and invasion 69. Li *et al.* 70 reported that miR-212 inhibited tumor growth by targeting SIRT1. SIRT1 is a highly conserved family of the class III histone deacetylase, commonly regulating the signaling axis by interacting with some genes involved 71. Luo *et al.* 72 reported that SIRT1 promoted autophagy and reduced hypoxia-induced apoptosis. Ramalinga *et al.* 73 showed that miR-212 down-regulation enhances autophagy by directly targeting SIRT1 in PCa cells, promoting angiogenesis and cellular senescence. It suggests a therapeutic potential of miR-212 for PCa.

#### MiR-205/TP53INP1 signaling

MiR-205, generally to be considered as a tumor suppressor, is reported to regulate the radiosensitivity of PCa cells by mediating the autophagy pathway 74. Bezawy *et al.* 75 reported that miR-205 reconstitution could significantly increases prostate response to radiotherapy. Similarly, tumor protein p53 inducible nuclear protein 1 (TP53INP1) is also a potential target of miR-205 in radiosensitivity regulation. Clinical data provide compelling evidence that autophagy contributes to both disease progression and therapeutic resistance in advanced PCa 76. Wang *et al.* 77 reported that miR-205 overexpression inhibited irradiation-induced autophagy in PCa by directly targeting TP53INP1 and substantially reduced the survival fraction of cells. However, TP53INP1 knockdown could suppress irradiation-induced autophagy and significantly enhance radiosensitivity in PCa cells 77. Moreover, restoring TP53INP1 substantially reversed the enhanced radiosensitivity induced by miR-205 overexpression 77. The miR-205/TP53INP1 mediated autophagy pathway may represent a novel therapeutic target for the treatment of PCa.

#### MiR-101/miR-17-92a/androgen receptor (AR) signaling

MiR-101 usually acts as a tumor suppressor in various malignancies, i.e., lung, gastric, liver, and colorectal cancer 78. In addition, it was reported that there was a close relationship between miR-101 and autophagy in multiple cancers 79. AR plays a key role in the growth of PCa cells and the progression of PCa. Celastrol has potential effects for treating PCa. Guo et al. revealed that AR could inhibit the celastrol-induced autophagy process through transactivation of miR-101 80. The authors found that the AR binding site is located in the upstream region of the miR-101 gene and highlighted that the miR-101-AR-autophagy axis might be a novel therapeutic target in PCa. Another study developed by Guo et al. showed that celastrol inhibited AR and its target miR-17-92a, resulting in autophagy induction in LNCaP cells 81. Collectively, the miR-101/miR-17-92a-AR axis involved autophagy played an important role in the development of PCa.

### Interaction of autophagy and miRNA in kidney cancer (Figure 2)

#### MiR-30a/Beclin-1 signaling

MiR-30a was dysregulated in several types of cancer and contributed to cancer carcinogenesis and progression. Jiang *et al*. 82 found that miR-30a was significantly down-regulated in renal cell carcinoma (RCC) tissues and cell lines as compared with adjacent non-cancerous tissues and normal renal cell lines. Moreover, re-expression of miR-30a could inhibit proliferation and migration of RCC cells 82. MiR-30a has been recognized as a potent inhibitor of autophagy by directly targeting Beclin-1 83. Meanwhile, autophagy activation induced by sorafenib was involved in chemo-resistance in RCC cells 84. Zheng *et al*. 85 demonstrated that miR-30a overexpression significantly inhibits autophagy activation and enhances sorafenib-induced cytotoxicity in RCC cells. These studies indicate that miR-30a may affect the effectiveness of sorafenib-mediated apoptosis via regulating autophagy, thus providing a novel strategy for treating RCC.

#### miR-335/Cyclin B1 (CCNB1) signaling

As a tumor suppressor or oncogene, miR-335 is down-regulated in multiple cancer tissues, and regulates the proliferation, invasion, and apoptosis of cancer cells. A recent study has shown that CCNB1 can be targeted by a specific miRNA and closely related to the occurrence and development of cancer 86. CCNB1 is a member of the cyclin family and triggers the G2/M transformation process via regulating CDK1 kinase, which may contribute to gene mutation or even tumor 87. Yan *et al*. 88 reported that the expression level of miR-335 in renal cancer tissues was lower than that in adjacent tissues. Also, inhibition of the miR-335/CCNB1 pathway promotes gemcitabine-induced autophagy and tumor growth, thus enhancing gemcitabine resistance in renal cancer 88. In addition, miR-335/CCNB1 overexpression can enhance gemcitabine sensitivity by inhibiting autophagy 88. Therefore, miR-335 may be a novel therapeutic target for the treatment of renal cancers with gemcitabine resistance.

#### miR-204/LC3B signaling

MiR204 is located in intron-9 of Transient Receptor Potential Melastatin 3 (TRPM3) on human chromosome 9. It was reported that several members of the TRPM family are implicated in multiple cancers. Since the genomic localization of miR-204 is within the TRPM3 gene, the TRPM3-miR-204 axis may play role in cancer development. Mikhaylova et al. 89 reported that miR-204 was almost lost in clear cell RCCs when compared to adjacent kidney tissues. And the authors further found that the VHL-regulated miR-204 could suppress RCC growth by inhibiting autophagy. MAP1LC3B (LC3B) was the direct target for miR-204. A subsequent study conducted by Hall et al. 90 revealed that TRPM3 could promote the growth of clear cell RCC and stimulate LC3A and LC3B autophagy, and the underlying mechanism was the VHL repressed TRPM3 expression via miR-204.

#### miR-100/NADPH oxidase 4 (NOX4)/mTOR signaling

MiR-100 was reported to serve as a promising prognostic marker for RCC. NOX4, a direct target gene of miR-100, is a sensor for oxygen, having the function of inhibition of tumor dissemination. A recent study 91 indicated that miR-100 could trigger autophagy and repress the invasion and migration of RCC cells by inhibiting the mTOR pathway via downregulating of NOX4 expression.

#### miR-143/K-RAS signaling

MiR-143 usually acts as an anti-oncomiR, suppressing the tumorigenesis in various types of cancers. K-RAS belongs to the RAS gene family members and have the function of encoding a small guanosine triphosphatase. Takai et al. 92 showed that ectopic expression of miR-143 might strengthen autophagy and G0/G1 cell-cycle arrest, thus remarkably inhibiting the growth of RCC cells. The authors further revealed that the mechanism was mainly based on miR-143 impairing K-RAS-signaling networks in RCC.

#### miR-429/ZEB1/JUN signaling

Long non-coding RNAs (lncRNAs) are one of the non-coding RNAs family. Secretory Carrier Membrane Protein 1 (SCAMP1) has been reported to be involved in the progression of various cancers. Additionally, miR-429 was proved to suppress the development of RCC via different mechanisms. Shao et al. 93 have conducted an *in-vitro* and *in-vivo* study and suggested that lncRNA SCAMP1 could regulate the expression of ZEB1/JUN as well as autophagy to promote the growth of pediatric RCC under oxidative stress through miR-429.

### Interaction of autophagy and miRNA in bladder cancer (Figure 3)

#### miR-222-PPP2R2A/Akt/mTOR axis

It was reported that elevated miR-222 levels are closely associated with a poor prognosis of bladder cancer. Zeng et al. 94 found that miR-222 plays a role in enhancing the proliferation of the T24 bladder cancer cell line. Cisplatin is the first-line treatment of chemotherapy in advanced bladder cancer. Zeng et al. also observed that miR-222 could attenuate cisplatin-induced cell death by inhibiting autophagy through activating the Akt/mTOR pathway. Further study revealed that blocking mTOR with rapamycin dramatically prevented miR-222-induced proliferation 94. Protein phosphatase 2A subunit B (PPP2R2A) is the direct target of miR-222, which commonly palys the role of a tumor suppressor. MiR-222 might modulate the PPP2R2A/Akt/mTOR axis to regulate the proliferation of bladder cancer cells and chemotherapeutic drug resistance.

#### MiR-221/TP53INP1/p-ERK axis

MiR-221 acts as an oncogene in various cancers, including bladder cancer by regulating autophagy. Tsikrika *et al.* 95 reported that bladder cancer patients with high expression of miR-221 have a higher short-term recurrence rate. Moreover, miR-221 overexpression has been reported to be an independent prognostic value for these patients' poor prognosis 95. It is reported that the tumor protein p53 inducible nuclear protein 1 (TP53INP1) is not only a regulator of autophagy but also a direct functional target of miR-221 96. Wang *et al.* 97 reported that autophagy acts in an inhibitory role in the initiation of bladder cancer. Liu *et al.* 98 found that the downregulation of miR-221 enhances autophagy activation via increasing TP53INP1 and inhibits migration and invasion of bladder cancer cells. In addition, TP53INP1 knockdown could partially abrogate the effect of the inhibition of miR-221 induced autophagy activation and suppression of cell invasion and migration 98.

#### miR-24-3p/death effector domain-containing protein (DEDD) axis

Differential expression of miR-24-3p is involved in many human diseases including bladder cancer 99. MiR-24-3p was down-regulated in PCa, while miR-24-3p overexpression significantly inhibited survival rate after the treatment of paclitaxel 100. DEDD belongs to the death effector domain-containing protein family and commonly carries out crucial roles in cell apoptosis and cell cycle. A recent study suggested that DEDD could reverse EMT via regulating selective autophagy 101. Yu *et al.* 102 reported that miR-24-3p increased autophagy by repressing DEDD, which promoted cell migration inhibiting apoptosis in bladder cancer. Based on this evidence, miR-24-3p may represent a pivotal potential therapeutic approach for the treatment of bladder cancer. On the other hand, Ye *et al.* 103 reported that miR-24-3p worked as a suppressor and might be recognized as a potential prognostic biomarker in nasopharyngeal carcinoma. However, the biological effect of miR-24-3p in bladder cancer was inconsistent with that in nasopharyngeal carcinoma. MiR-24-3p was overexpressed in bladder cancer and promoted cell proliferation, migration, and invasion of the cancer cells 104.

#### miR-139-5p/Bmi-1/AMPK/mTOR pathway

It was reported that miR-139-5p expression could predict the prognosis of multiple cancers, while increased miR-139-5p indicated a worse prognosis in patients. Luo et al. 105 suggested that miR-139-5p inhibited the proliferation capability in bladder cancer by directly targeting Bmi-1. A recent study that investigated bladder cancer also indicated that Bmi-1 was the target protein of miR-139-5p. It was suggested that sodium butyrate (NaB) has antitumor effects in multiple human cancer cells. Bmi-1 plays an important role in maintaining mitochondrial function and reactive oxygen species (ROS) homeostasis. Depletion of Bmi-1 genetically often caused ATP reduction and AMPK-activated autophagy 106. Wang et al. 107 demonstrated that NaB inhibited migration and induced AMPK-mTOR pathway-dependent autophagy and ROS-mediated apoptosis through the miR-139-5p/Bmi-1 axis in bladder cancer cells. AMPK/mTOR pathway could activate autophagy and mitochondrial dysfunction in bladder cancer cells.

## The therapeutic potentials related to the interference of miRNA-autophagy related mechanisms

According to the current evidence, it was suggested that microRNA-mediated regulation of autophagy can influence the sensitivity of urological cancer cells to radiotherapy and chemotherapy. Autophagic activation is a response of tumor cells to radiotherapy or chemotherapy 22, 108. In most of the cases, abnormal autophagy may lead to carcinogenesis, promoting tumor cells to become resistant to radiotherapy or chemotherapy 109. This action may cause by autophagy which providing the energy required for evade apoptosis induced by radiotherapy and chemotherapy, leading to treatment resistance 110. microRNAs have been found to act as major autophagy regulatory factors affecting radiotherapy/chemotherapy resistances, including urologic malignancies 111, 112. For example, Liu et al. reported that miR-143 might sensitize the PCa cells (PC3 and DU145) to radiation by inhibiting autophagy. Consistent with this finding, Wang et al. 77 found that miR-205 overexpression significantly suppressed irradiation-induced autophagy in PCa cells and thus reduced the survival fraction of the cancer cells. As for renal cell carcinoma, Yan et al. 88 demonstrated that hsa-circ_0035483 binds miR-335 to enhance gemcitabine resistance by elevating autophagic level. Clinically, miRNA-mediated autophagy also play role in bladder cancer progression. Zeng et al. 94 showed that has-miR-222 dramatically attenuated chemotherapeutic drug-induced cell death in bladder cancer cells through the inhibition of autophagy. These findings provide a novel insight into the role of miRNA-mediated autophagy in urologic oncology radiotherapy and chemotherapy. Aberrant urologic cancer-related miRNAs may concur with autophagy to determine the cancer cells response to radiation and drug therapy, which is a key field needing further investigation.

## Limitations and future perspectives

**Figure 4:**
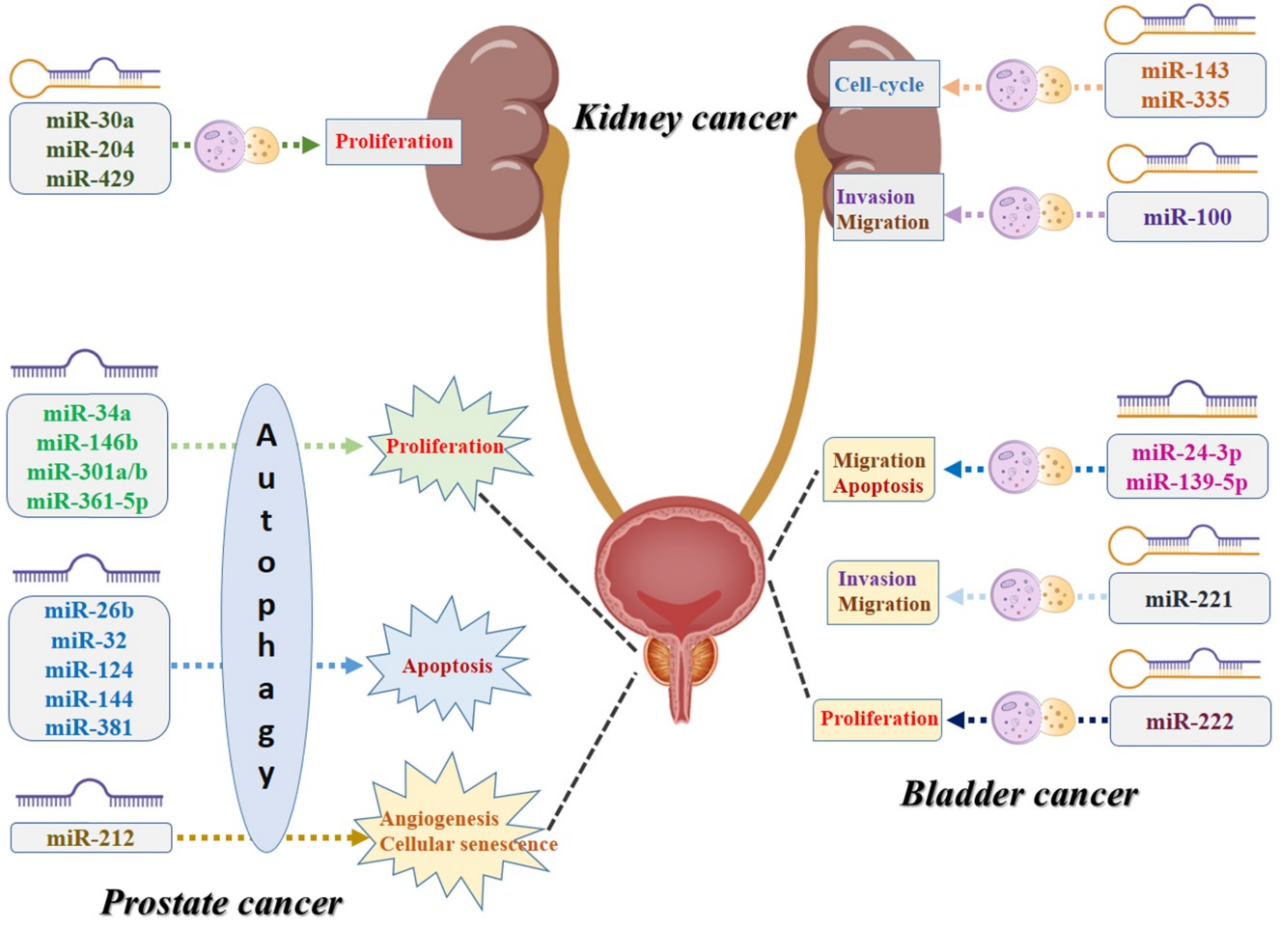
Schematic diagram of the biological mechanisms underlying miRNA-associated autophagy in the development of urologic cancers.

To our knowledge, this is the first study that performs a systematic review to summarize all the evidence of the association between miRNA and autophagy in urologic oncologies. As shown in Figure 4, numerous specific miRNAs-mediated autophagy may play a crucial role in malignant transformation of the urologic cancers, affecting the capability of proliferation, apoptosis, cell-cycle, migration, invasion, angiogenesis, and cellular senescence of the cancer cells. For prostate cancer, miR-34a, miR-146b, miR-301a/b, and miR-361-5p-mediated autophagy play their roles in cancer development via affecting the proliferation capability of the cancer cells, while miR-26b, miR-32, miR-124, miR-144, and miR-381 and their corresponding autophagic process are associated with the process of apoptosis. For kidney cancer, miR-30a, miR-204, and miR-429 can regulate the cancer cell proliferative potential by acting directly/indirectly with autophagy, while miR-143 and miR-335-mediated autophagy are believed to contribute to tumor growth by affecting the cell-cycle. For bladder cancer, miR-24-3p and miR-139-5p-associated autophagy influence tumor development mostly by regulating migration and apoptosis, while miR-221 has been reported to be related to invasion and migration and miR-222 may correlate to the variations of the proliferative potential of the cancer cells. However, several inherent limitations should be acknowledged when interpreting this comprehensive review. First, the diagnostic, therapeutic, and prognostic value of the coefficient of miRNA and autophagy in urologic cancers still need to be further investigated due to limited clinical studies. Second, the molecular mechanisms of miRNA-mediated autophagy and urologic tumorigenesis must be further clarified and explored. In addition to the targeting genes and the corresponding signaling pathways, some other contributors, such as tumor microenvironment and tumor immunity, might also play roles in the pathomechanism and development of urologic oncologies. Third, we should note that autophagy plays dual roles in cancer, acting not only as a tumor suppressor but also promoting tumorigenesis in specific urologic oncologies. Once such pathogenesis is addressed, it is conducive to the discovery of some valuable diagnostic and prognostic biomarkers and innovative therapeutic alternatives in urologic malignancies. MiRNA-mediated autophagy plays an important role in tumorigenesis, progression, and resistance to anticancer therapies. Given that, targeting the aforementioned miRNA for autophagy modulation may present as the reliable diagnostic and prognostic biomarkers or promising therapeutic strategies in urologic oncologies.

## Conclusion

Based on this review, miRNA-associated autophagy could be a critical molecular mechanism in the initiation and progression of urologic oncologies, such as PCa, bladder cancers, and kidney cancers. Upregulation or downregulation of miRNAs could activate or inhibit autophagy in these cancers. MiRNA targeted genes and the different signaling pathways constitute a complex network that orchestrates autophagy regulation, militating the oncogenic and tumor-suppressive effects in urologic malignancies.

## Figures and Tables

**Figure 1:**
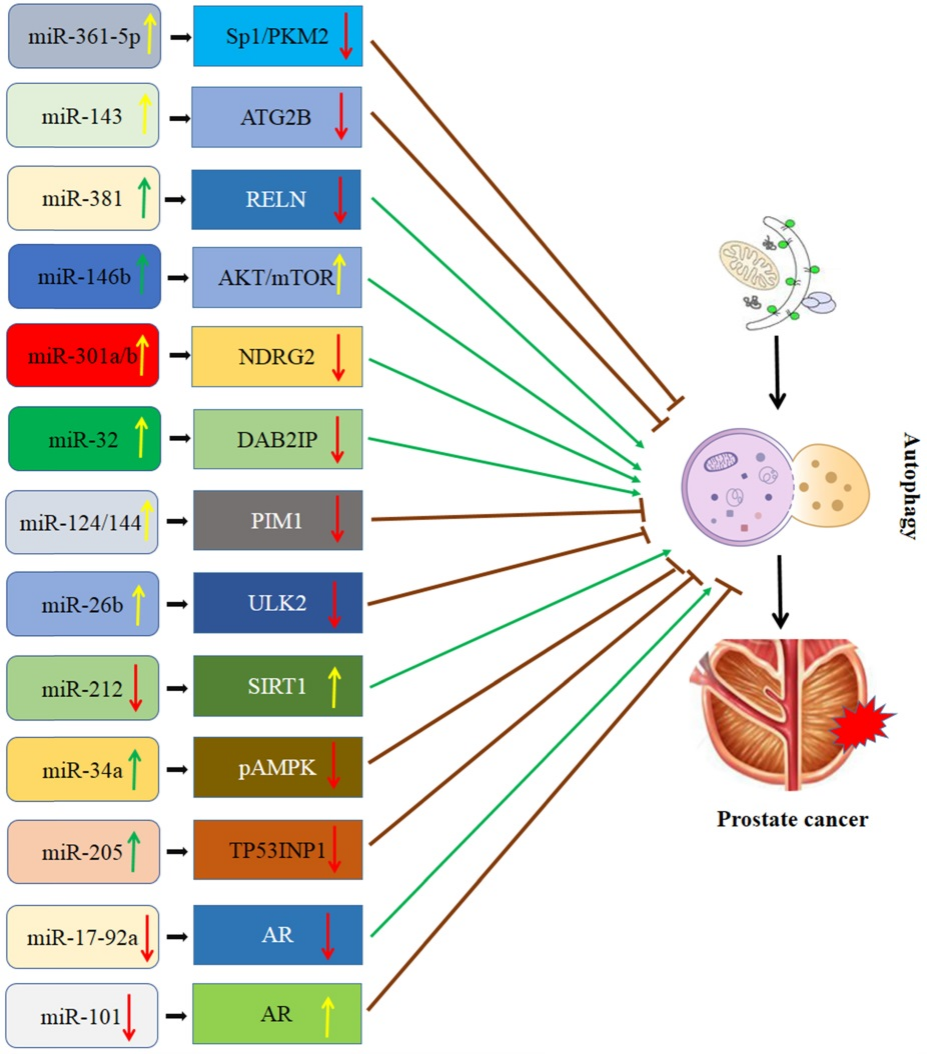
Schematic diagram of the association between miRNA-mediated autophagy in prostate cancer. AR: androgen receptor; ATG2B: autophagy-related 2B; DAB2IP: DAB2 interacting protein; NDRG2: N-myc downsream regulator gene 2; PIM1: Polymers of intrinsic microporosity-1; SIRT1: Sirtuin 1; Sp1/PKM2: RELN: Reelin; Specificity protein 1/pyruvate kinase isoenzyme type M2; TP53INP1: Tumor protein p53 inducible nuclear protein 1; ULK2: Unc-51 like kinase 2.

**Figure 2:**
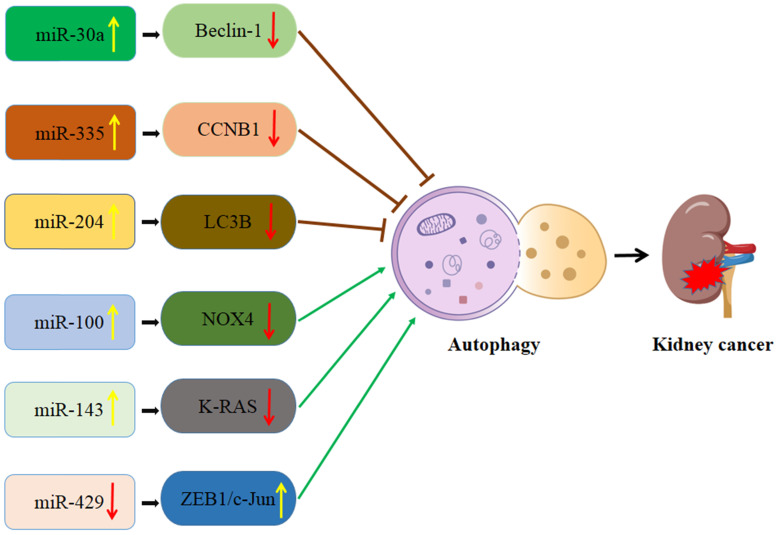
Schematic diagram of the association between miRNA-mediated autophagy in kidney cancer. CCNB1: Cyclin B1; LC3B: MAP1LC3B; NOX4: NADPH oxidase 4.

**Figure 3:**
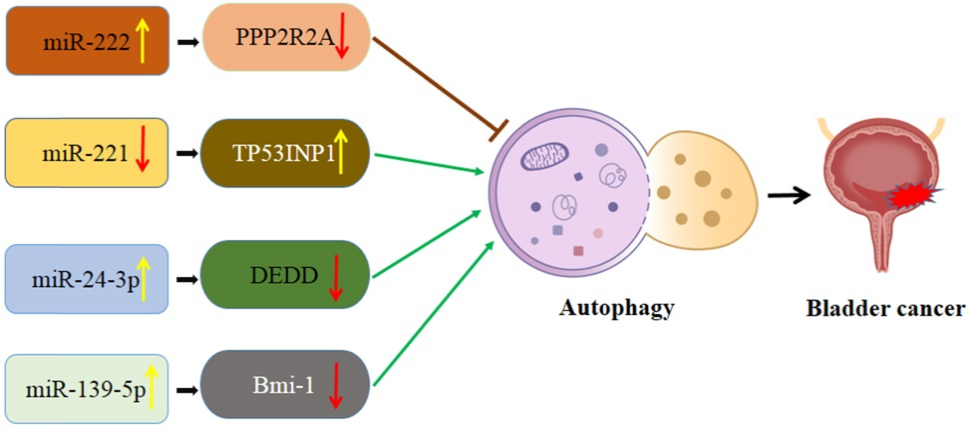
Schematic diagram of the association between miRNA-mediated autophagy in bladder cancer. PPP2R2A: TP53INP1: Protein phosphatase 2A subunit B; tumor protein p53 inducible nuclear protein 1; DEDD: death effector domain-containing protein; Bmi-1: B cell‑specific Moloney murine leukemia virus integration site 1.

**Table 1:** The Role of Autophagy Modulated by MiRNAs in Cancer Initiation and Cancer Development

Cancer Specificity	MiRNAs	oncomiRNA/tsmiRNA	Target	Anti-/Proautophagy	References
Prostate cancer	miR-26b	tsmiRNA	ULK2	Antiautophagy	65,66
	miR-32	oncomiRNA	DAB2IP	Proautophagy	57
	MiR-34a	tsmiRNA	pAMPK	Antiautophagy	49,50
	miR-101	tsmiRNA	AR	Proautophagy	80
	miR-124/144	tsmiRNA	PIM1	Antiautophagy	62
	miR-143	tsmiRNA	ATG2B	Antiautophagy	47
	miR-146b	oncomiRNA	AKT/mTOR	Proautophagy	52
	miR-205	tsmiRNA	TP53INP1	Antiautophagy	77
	miR-212	tsmiRNA	SIRT1	Antiautophagy	73
	miR-301a/b	oncomiRNA	NDRG2	Proautophagy	53,55
	miR-361-5p	tsmiRNA	Sp1/PKM2	Antiautophagy	45
	miR-381	tsmiRNA	RELN	Proautophagy	51
	miR-17-92a	oncomiRNA	AR	Antiautophagy	81
Renal cancer	MiR-30a	tsmiRNA	Beclin-1	Antiautophagy	83,85
	MiR-100	tsmiRNA	NOX4	Proautophagy	91
	miR-143	tsmiRNA	K-RAS	Proautophagy	92
	MiR-204	tsmiRNA	LC3B	Antiautophagy	89,90
	MiR-335	tsmiRNA	CCNB1	Antiautophagy	88
	miR-429	tsmiRNA	ZEB1/JUN	Antiautophagy	93
Bladder cancer	miR-24-3p	oncomiRNA	DEDD	Proautophagy	102,104
	miR-139-5p	tsmiRNA	Bmi-1	Proautophagy	105,107
	miR-221	oncomiRNA	TP53INP1	Proautophagy	96-98
	miR-222	oncomiRNA	Akt/mTOR	Antiautophagy	94

## Abbreviations

AR: androgen receptor
ATG2B: autophagy-related 2B
Bmi-1: B cell‑specific Moloney murine leukemia virus integration site 1
CCNB1: Cyclin B1
DAB2IP: DAB2 interacting protein
DEDD: death effector domain-containing protein
LC3B: MAP1LC3B
NDRG2: N-myc downsream regulator gene 2
NOX4: NADPH oxidase 4
PIM1: Polymers of intrinsic microporosity-1
PPP2R2A: Protein phosphatase 2A subunit B
SIRT1: Sirtuin 1
RELN: Reelin
Sp1/PKM2: Specificity protein 1/pyruvate kinase isoenzyme type M2
TP53INP1: Tumor protein p53 inducible nuclear protein 1
ULK2: Unc-51 like kinase 2
